# Functional status and quality of life 12 months after discharge from a medical ICU in healthy elderly patients: a prospective observational study

**DOI:** 10.1186/cc10121

**Published:** 2011-03-28

**Authors:** Emilio Sacanella, Joan Manel Pérez-Castejón, Josep Maria Nicolás, Ferran Masanés, Marga Navarro, Pedro Castro, Alfonso López-Soto

**Affiliations:** 1 Geriatric Unit, Department of Internal Medicine Hospital Clínic of Barcelona, Villarroel, 170, Barcelona 08036, Spain; 2 Institut d'Investigacions Biomèdiques August Pi i Sunyer (IDIBAPS), Faculty of Medicine, University of Barcelona, Casanova 143, Barcelona 08036, Spain; 3 Intensive Care Unit, Department of Internal Medicine Hospital Clínic of Barcelona, Villarroel, 170, Barcelona 08036, Spain

## Abstract

**Introduction:**

Long-term outcomes of elderly patients after medical ICU care are little known. The aim of the study was to evaluate functional status and quality of life of elderly patients 12 months after discharge from a medical ICU.

**Methods:**

We prospectively studied 112/230 healthy elderly patients (≥65 years surviving at least 12 months after ICU discharge) with full functional autonomy without cognitive impairment prior to ICU entry. The main diagnoses at admission using the Acute Physiology and Chronic Health Evaluation III (APACHE III) classification diagnosis and length of ICU stay and ICU scores (APACHE II, Sepsis-related Organ Failure Assessment (SOFA) and OMEGA) at admission and discharge were collected. Comprehensive geriatric assessment included the presence of the main geriatric syndromes and the application of Lawton, Barthel, and Charlson Indexes and Informant Questionnaire on Cognitive Decline to evaluate functionality, comorbidity and cognitive status, respectively. The EuroQol-5D assessed quality of life. Data were collected at baseline, during ICU and ward stay and 3, 6 and 12 months after hospital discharge. Paired or unpaired T-tests compared differences between groups (continuous variables), whereas the chi-square and Fisher exact tests were used for comparing dichotomous variables. For variables significant (*P *≤ 0.1) on univariate analysis, a forward multiple regression analysis was performed.

**Results:**

Only 48.9% of patients (mean age: 73.4 ± 5.5 years) were alive 12 months after discharge showing a significant decrease in functional autonomy (Lawton and Barthel Indexes) and quality of life (EuroQol-5D) compared to baseline status (*P *< 0.001, all). Multivariate analysis showed a higher Barthel Index and EQ-5D _vas _at hospital discharge to be associated factors of full functional recovery (*P *< 0.01, both). Thus, in patients with a Barthel Index ≥ 60 or EQ-5D _vas _≥40 at discharge the hazard ratio for full functional recovery was 4.04 (95% CI: 1.58 to 10.33; *P *= 0.005) and 6.1 (95% CI: 1.9 to 19.9; *P *< 0.01), respectively. Geriatric syndromes increased after ICU stay and remained significantly increased during follow-up (*P *< 0.001).

**Conclusions:**

The survival rate of elderly medical patients 12 months after discharge from the ICU is low (49%), although functional status and quality of life remained similar to baseline in most of the survivors. However, there was a two-fold increase in the prevalence of geriatric syndromes.

## Introduction

Admission of elderly patients to the intensive care unit (ICU) occurs frequently in Western countries [1,2] and this situation will probably grow in the near future [2]. In spite of this, many physicians have doubts as to whether elderly subjects are good candidates for ICU care because of the apparently, albeit possibly false, poor long-term outcomes (such as mortality, functional autonomy and quality of life) after critical care in this population [3-12]. It is especially important to confirm or to rule out this hypothesis in healthy elderly subjects with a good pre-morbid status before ICU admission and who have a theoretical long life expectancy prior to critical care admission (up to 20 years) [13]. Indeed, to our knowledge no previous study has been focussed on this specific population of elderly medical patients.

Up to now, several papers have evaluated the outcomes of elderly subjects after ICU care; however, the results obtained have been very heterogeneous [1-3,5,7]. In addition, some intensive care physicians have suggested that lower treatment intensity in elderly compared to younger subjects could be the cause of worse outcomes in these individuals [4] with recent higher treatment intensity applied to older subjects having been associated with better outcomes [14].

Short- and long-term mortality of elderly patients after ICU care is reported to be between 11 to 38% and 22 to 69%, respectively, whereas functional autonomy and quality of life may be moderately decreased in 10 to 60% of subjects [1,2,5,12,15]. This great heterogeneity in the results obtained may be due to significant differences in the methodology used and also in the patients (age, pre-morbid status, main diagnosis at ICU entry) and the type of ICU (surgical or medical) studied, making it difficult to obtain conclusions about the outcomes of specific subpopulations of elderly subjects after ICU discharge [3,6,8-10,16]. Boumendil *et al*. [1] recently suggested that to answer this question, specific groups of critically ill elderly patients should be prospectively studied to identify those with a better prognosis.

Most studies on the outcome of elderly patients after ICU care have been restricted mainly to surgical patients [6,8,9,11], although medical ICU patients are known to usually have a worse prognosis [3,6,8,9]. Thus, in a recent series on this issue only 11 subjects (5%) were medical patients [11]. In summary, the results in the literature are heterogeneous, making it difficult to achieve recommendations for decision making related to the admission of elderly subjects to the ICU, especially in those with a good pre-morbid status with a medical condition.

Therefore, we embarked on a prospective observational study in a series of healthy community-dwelling elderly patients with a good pre-morbid status prior to ICU admission to evaluate the long-term outcomes in terms of functional and cognitive status and quality of life after non-elective medical ICU admission.

## Materials and methods

### Patient selection

**Figure 1:**
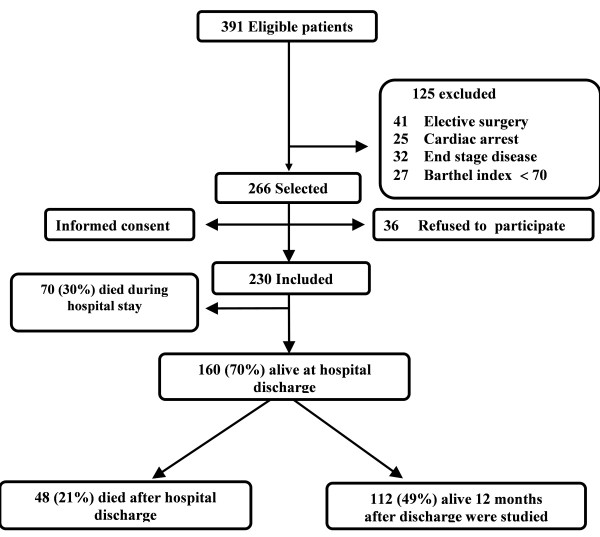
**Flow Chart of the Patients Studied**. Flow chart of the patients eligible for the study and those finally studied during follow-up.

We performed a prospective observational study in an eight-bed medical ICU. We enrolled patients ≥65 years living at home with full autonomy (Barthel Index (BI) ≥70), without cognitive impairment, and who were non-electively admitted to the ICU for a medical condition. Patients admitted to the ICU after cardiac arrest or with end-stage disease were excluded. A total of 230 patients were enrolled. Of these, 160 were discharged alive from hospital, 48 of whom died after discharge and the remaining 112 patients (49%) were alive one year later and were evaluated as described below (Figure 1). All the patients were enrolled in the first 24 to 48 hours after ICU admission. The participants or a close relative gave informed consent to participate in the study which was performed in accordance with the ethical standards established in the 1964 Declaration of Helsinki. The institutional review board of Hospital Clínic, Barcelona, Spain, approved the study protocol.

### ICU data

The main diagnoses at admission were collected using the Acute Physiology and Chronic Health Evaluation III (APACHE III) classification diagnosis and length of ICU stay. Severity of illness, organ dysfunction and therapeutic intensity were measured using the APACHE II [17], Sepsis-related Organ Failure Assessment (SOFA) [18] and OMEGA [19] scores, respectively. The OMEGA score is used to assess therapeutic intensity in the ICU and is constituted of 47 diagnostic and therapeutic parameters each with an assigned value; the total OMEGA score is obtained by the sum of these values throughout ICU stay.

### Comprehensive geriatric assessment (CGA) and Quality of Life (QOL) evaluation

A CGA including functional, neuropsychological, comorbidity, QOL evaluation and geriatric syndromes assessment were performed in all the patients. Functional evaluation included assessment of autonomy in instrumental and basic activities of daily life (IADL and ADL, respectively) which were evaluated with the Lawton Index (LI) [20] and Barthel Index [21], respectively. Both are quantitative scales ranging from 0 to 8 (LI) and from 0 to 100 (BI). Scores of 8 and 100 points, respectively, denote full autonomy in IADL and ADL. Cognitive function was assessed with the Informant Questionnaire on Cognitive Decline in the Elderly (IQCODE) or the Minimental Status Evaluation (MMSE) when possible; the Charlson Index was used to evaluate comorbidity [22]. We considered the baseline status of the patient (in terms of functionality and quality of life) as the situation that the patient had before becoming ill and subsequently requiring ICU admission.

Finally, quality of life was measured using the EuroQol-5D (EQ-5D), a health status scale validated in critical patients in our country [23]. This scale evaluates five domains (mobility, self-care, usual activities, pain/disorder and anxiety/depression) and includes the EQ-5D index and the EQ-5D vas, a visual analogue scale ranging from 0 to 100. All the scales were always completed by the patients. Nonetheless, a close relative was interrogated when the patient was unable to answer on the baseline visit. Concordance between self-administered and family member-administered scores was evaluated with the Kappa Index (KI). The KI was high (0.71 to 0.83) when mobility, personal care, usual activities and global health status (good vs. bad health) were evaluated, being slightly lower (0.55 to 0.62) when anxiety/depression or pain/discomfort was assessed.

### Follow-up studies

The CGA evaluation was performed at baseline, at ICU and hospital discharge and also 3, 6 and 12 months after hospital discharge. On every scheduled appointment in our out-patient geriatric clinic a member of the geriatric team performed the CGA evaluation with the validated scales described above. Likewise, assessment of the incidence/prevalence of the main geriatric syndromes (urinary incontinence, faecal incontinence, depression, delirium, falls, immobility, cognitive impairment, polypharmacy and malnutrition) was also performed.

Good outcome was described as a decrease in the Lawton Index (≤2 points) and/or Barthel Index (≤20 points) and/or EQ-5D _vas _(≤20 points), compared to pre-ICU status.

### Diagnostic criteria for main geriatric síndromes

The term ''geriatric syndrome'' refers to common clinical conditions in older persons that do not fit into specific disease categories. Delirium (assessed by the Confusion Assessment Method score), falls (two or more in the last six months), immobility, pressure ulcers, malnutrition, cognitive impairment (abnormal scoring in MMSE or IQCODE), polypharmacy (4 ≥drugs/day), depression (the Yesavage score was applied) [24] and urinary and or faecal incontinence are classified as geriatric syndromes. These conditions are highly prevalent and multifactorial and are associated with substantial morbidity, poor outcomes and worse quality of life in elderly subjects [25].

### Statistical analysis

The data were analysed using SPSS-PC 16.0 statistical software (SPSS, Chicago, IL, USA). As almost all variables followed a normal distribution, variables were expressed as mean ± standard deviation. For continuous variables paired or unpaired T-tests were used to compare differences between groups, whereas the chi-square and Fisher exact tests were used to compare dichotomous variables. In addition, we used an ANOVA for multiple comparisons when appropriate. A two-tailed *P-*value < 0.05 was considered statistically significant. Forward multiple regression analysis (in: 0.05; out: 0.10) was performed in variables which were significant (*P *≤ 0.1) on univariate analysis (age, length of ICU stay, BI, EQ-5D _vas _and geriatric syndromes at hospital discharge).

## Results

### Demographic data

The main features of the initial cohort (*n *= 230) were: mean age: 74.5 ± 5.6 years; APACHE score at ICU entry: 19.7 ± 5.7 points; mean ICU stay: 11.7 ± 11.6 days; 71 and 7% of patients underwent mechanical ventilation and haemodialysis, respectively; and hospital mortality (ICU + Ward) of 30%. A detailed description of the baseline characteristics and mortality (in- and out-hospital) of the initial cohort has been published elsewhere [15].

Of the 160 patients alive after ICU care, 48 died during the following months after hospital discharge, thus only 112 subjects remained alive one year later. The mean age of this subcohort (*n *= 112) was 73.4 ± 5.5 years (range: 65 to 87 years), thus, 74 years was the cut-off point used to classify patients as young-old (65 to 74 years; *n *= 62 patients, 55%) and old-old (≥75 years; *n *= 50 patients, 45%). Ninety-eight percent of patients were living at home until the day prior to ICU admission.

### Baseline characteristics of the patients

Table 1 shows the main baseline characteristics of the patients including ICU data and functional status prior to intensive care admission. The mean APACHE II at ICU entry was 19.2 ± 6 points (range 8 to 47 points) and the length of ICU stay was 9.4 ± 10.2 days (range 2 to 54 days). Mechanical ventilation and haemodialysis were applied in 54 and 4% of patients, respectively. All the patients showed an excellent baseline functional status evaluated by the Barthel and Lawton Indexes. Indeed, only 5% of patients had a basal Barthel Index <85 points. In addition, cognitive status assessed with the IQCODE was normal in 98% of the subjects whereas comorbidity was moderate. Older subjects had a slightly decreased Lawton Index compared to younger patients (*P *= 0.016) whereas women had a lower Charlson Index (Table 1).

**Table 1:** Demographic, ICU and comprehensive geriatric assessment data at baseline

	Whole group of patients (*n *= 112)	Patients 65 to 74 years old (*n *= 62)	Patients ≥75 years (*n *= 50)
Age (y)	73.4 ± 5.5	69.3 ± 2.8	78.5 ± 3.1
Sex (M/F)%	57/43	60/40	54/46
APACHE II at ICU admission	19.2 ± 6.0	19.1 ± 4.9	19.2 ± 7.1
SOFA at ICU admission	5.6 ± 3.7	5.7 ± 3.9	5.4 ± 3.5
Length of ICU stay (d)	9.4 ± 10.2	10.7 ± 11.9	7.9 ± 7.3
Cardiac disease n, (%)	13 (12)	5 (10)	8 (16)
Respiratory disease n, (%)	49 (44)	23 (37)	26 (52)
Severe sepsis/septic shock n, (%)	23 (20)	14 (23)	9 (18)
Cerebrovascular disease n, (%)	13 (12)	9 (14)	4 (8)
Other medical disease n, (%)	14 (12)	10 (16)	4 (8)
Mechanical ventilation n, (%)	62 (54)	28 (45)	34 (68)*
Haemodialysis/CVVHDF n, (%)	4 (3, 6)	1 (1, 6)	3 (6)
OMEGA score	115.3 ± 122.8	132.2 ± 151.1	95.3 ± 74.1
Lawton Index	6.8 ± 1.6	7.2 ± 1.4	6.4 ± 1.7*
Barthel Index	96.4 ± 8.7	96.6 ± 9.3	96.1 ± 7.9
Charlson Index	2.4 ± 1.7	2.2 ± 1.6	2.7 ± 1.8
EQ-5D _vas_	76.1 ± 16.4	78.0 ± 15.9	73.2 ± 16.8
EQ-5D _index _= 11,111 (%)	38	40	35

*P *< 0.05 vs. young-old patients.

Acute Physiology and Chronic Health Evaluation (APACHE).

Sepsis-related Organ Failure Assessment (SOFA).

Continuous venovenous haemodiafiltration: CVVHDF.

EuroQol-5D visual analogue scale: EQ-5D _vas_; EuroQol-5D _index_: EQ-5D _index_.

The quality of life before ICU admission was good with no differences between younger and older subjects. Thus, three quarters of the patients had 70 or more points in the EQ-5D _vas _which is the level considered as good health. Up to 65% of the subjects had slight impairment in only one of the EQ-5D subdomains, being pain/discomfort (37.9%) the most affected. Only 21% of subjects, especially the more elderly (*P *= 0.004), had two or more geriatric syndromes at baseline being polypharmacy, falls and depression the most prevalent. The presence of geriatric syndromes (≥2) was directly associated with a lower perceived quality of life assessed by EQ-5D _vas _(64.6 ± 17.3 vs. 79.7 ± 15.6; *P *= 0.002) and also with a worse functional status in IADL and ADL *P *< 0.05, both).

### Functional status and quality of life during follow-up

**Figure 2:**
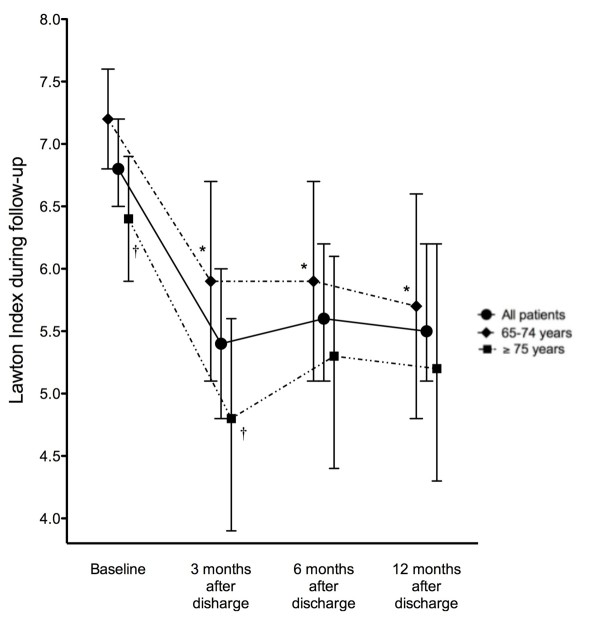
**Changes in mean Lawton Index (mean, 95% CI) during Follow-up**. Functional status in instrumental activities of daily living measured by the Lawton Index (range 0 to 8) during the follow-up period in the whole group and also in young-old and old-old patients separately. * *P *< 0.01 compared to baseline status in younger and older subjects. † *P *< 0.01 compared to younger subjects.

**Figure 3:**
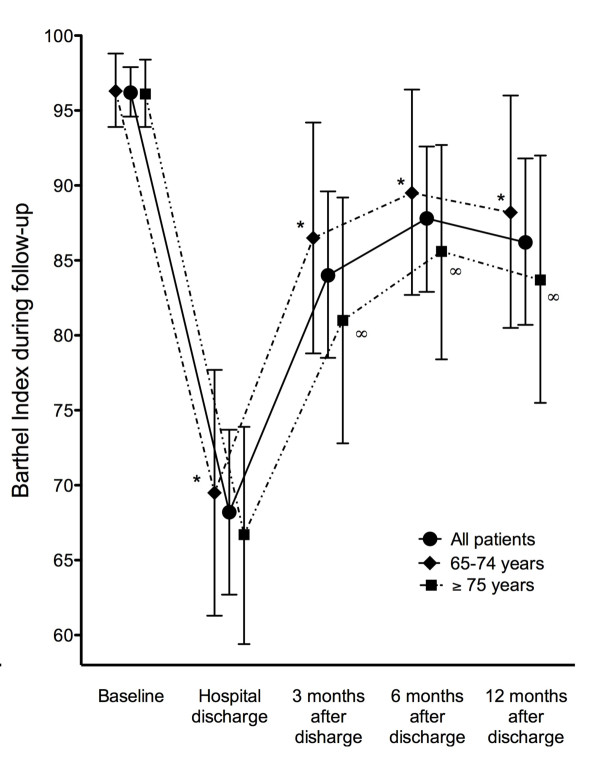
**Changes in mean Barthel Index (mean, 95% CI) during follow-up**. Functional status in basic activities of daily living measured by the Barthel Index (range 0 to 100) during the follow-up period in the whole group and also in young-old and old-old patients separately. * *P *< 0.01 compared to baseline BI (younger and older subjects). ∞ *P *< 0.01 compared to BI at hospital discharge (younger and older subjects).

Autonomy in IADL was significantly decreased after discharge (*P *< 0.001) and the baseline situation in IADL was not recovered in the following 12 months (6.7 ± 1.7 vs. 5.3 ± 2.6 points; *P *< 0.001) (Figure 2). In fact, the previous Lawton Index was not achieved in up to 45% of the subjects at the end of the study period, with no significant differences between younger and older subjects. However, a significant decrease was observed in IADL autonomy (decrease in ≥2 activities) in only 27% of patient whereas the IADL autonomy improved in 6%. Likewise, the whole cohort (also both groups separately) showed a significant decrease in ADL autonomy (96.3 ± 8.8 vs. 69.8 ± 29.2 points) at hospital discharge (*P *< 0.001 both) which was not fully recovered in the following 12 months (96.3 ± 8.8 vs. 87.1 ± 22.8; *P *< 0.001) (Figure 3). In fact, up to 37% of the patients did not achieve their previous Barthel Index at the end of the study period. However, a significant decrease in ADL autonomy (decrease in ≥20 points in BI) was only observed in 17% of patients whereas 11% of subjects improved. The patients who did not achieve full recovery were of 75 years of age or more (55 vs. 22%; *P *= 0.002). Maximal functional recovery was achieved in the first three to six months after discharge without additional improvement in the following six-month period and autonomy in IADL and ADL was similar in older and younger subjects at the end of the follow-up (Figures 2 and 3). Functional status (LI and BI) during post-hospital follow-up was not significantly influenced by the main diagnosis at ICU entry or by the use of mechanical ventilation. However, patients with an OMEGA score greater than 67 (the 50^th ^percentile of the cohort) had a lower Barthel Index at hospital discharge and 3, and 12 months after discharge compared to those with an OMEGA score lower than 67 (*P *< 0.05, all).

Cognitive Status was normal in 85% of the patients at hospital discharge whereas 15% (*n *= 17) had a MMSE <24 points. One third did not recover during follow-up. At the end of the study, 10% of patients had a score lower than 24 in the MMSE, half of the patients had an abnormal MMSE score after hospital discharge and cognitive impairment developed during follow-up in the other half.

At hospital discharge the EQ-5D _vas _was significantly lower compared to baseline, 55.5 ± 19.6 vs. 76.1 ± 16.4 points (*P *< 0.001), and progressively improved in the following months. However, the EQ-5D _vas _remained lower than at baseline (67.9 ± 16.8 vs. 76.1 ± 16.4; *P *= 0.034) 12-months after discharge. Indeed, at the end of follow-up 61% of patients had a lower EQ-5D _vas _compared to that obtained at the beginning of the study, although a clinically relevant (≥20 points) decrease in this score was only observed in a minority of the patients (31%). In addition, we observed that only 18% of the subjects reported no disability in any EQ domains 12-months after discharge compared to 38% at baseline. However, most of the disabilities (75%) were of slight or moderate intensity. In fact, only 17% of the patients had a decrease of four or more points in EQ-domains whereas up to 83% of patients improved or had minor changes in EQ-domains during follow-up. Anxiety, pain and usual activities were the domains most frequently affected at the end of the study.

### Geriatric syndromes during follow-up

**Figure 4:**
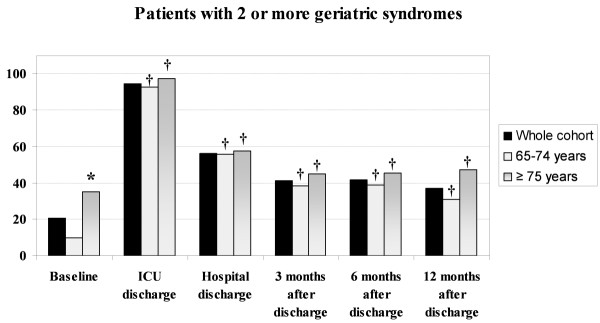
**Percentage of Patients with Two or More Geriatric Syndromes at Each Scheduled Evaluation**. Prevalence of ≥2 geriatric syndromes at each scheduled evaluation in the whole group and also in young-old and old-old patients separately. * *P *< 0.001, compared to younger patients. † *P *< 0.05 compared to baseline status.

The prevalence of subjects with ≥2 geriatric syndromes increased immediately after ICU admission and up to 95% at ICU discharge, and decreased slowly thereafter. Nonetheless, this prevalence remained higher 12-months after discharge (37.2%) compared to baseline (*P *< 0.001) (Figure 4). Polypharmacy 70.8%, urine incontinence 23%, depression 18.8%, immobility 16.7%, faecal incontinence 13% and cognitive impairment 10% were the most frequent geriatric syndromes at the end of follow-up.

Finally, as expected, the QOL, IADL and ADL autonomy of the subjects with two or more geriatric syndromes was worse than those with less than two geriatric síndromes (*P *< 0.01, all).

### Factors associated with good long-term outcome

Only 112 (48.7%) of 230 patients of the initial cohort were alive 12 months after hospital discharge. In these 112 subjects, a higher Barthel Index (*P *= 0.001), higher EQ- 5D_vas _(*P *< 0.03) and fewer geriatric syndromes at hospital discharge (*P *< 0.01) were predictors of full functional recovery in the following months (univariate analysis).

Thus, 71% of patients with a Barthel Index ≥ 60 at hospital discharge achieved full recovery in ADL compared to 39% with a Barthel Index <60 (*P *= 0.005). Subjects with a Barthel Index ≥60 at hospital discharge had a hazard ratio for full functional recovery in ADL of 4.04 (95% CI: 1.58 to 10.33; *P *= 0.005) compared to those with a lower Barthel Index at that time. On the other hand 69% of subjects with EQ-5D _vas _≥ 40 at hospital discharge achieved full recovery in IADL compared to 26% with an EQ-5D_vas _<40 (*P *< 0.01). Thus, patients with EQ-5D_vas _≥ 40 at hospital discharge had a hazard ratio for full recovery in IADL of 6.1 (95% CI: 1.9 to 19.9; *P *< 0.01]. On multivariate analysis the predictive factor for full recovery in ADL and IADL was the Barthel Index and EQ-5D _vas _at hospital discharge, respectively (both, *P *= 0.001).

### Patient death during post-hospital follow-up

As shown in Figure 1, a group of patients (*n *= 48, mean age of 76 ± 5 years) died shortly after hospital discharge (median survival time: 45 days). These patients were significantly older (*P *= 0.006), more frequently mechanically ventilated (71 vs. 54%; *P *= 0.035) and had a lower Lawton Index (*P *= 0.012), EQ-5D _vas _(*P *= 0.004) as well as a greater number of geriatric syndromes at baseline (*P *= 0.019) compared to patients with a long-survival time. However, no differences were observed in ICU scores (APACHE, SOFA and OMEGA), length of ICU stay, Charlson Index, main diagnostic categories at ICU entry and the Barthel Index at ICU admission and at hospital discharge between the two groups.

## Discussion

Healthy elderly patients have a low survival rate 12 months after discharge from a medical ICU. In patients who survive at least one year, more than two thirds have a similar functional autonomy and quality of life compared to baseline, although there is a two-fold increase in the prevalence of the main geriatric syndromes. Functional status (Barthel Index) and quality of life (EQ-5D) at hospital discharge are the best predictive factors for full functional recovery at long-term follow-up.

The elderly population is growing in the ICUs in Western countries with patients aged 75 or more years representing 20 to 25% of the total ICU patients at the beginning of the 21st century compared to 12% in the late 1990s [1,2,5]. A recently published study performed in Australia and New Zealand calculated a potential increase of 72% in ICU demand for patients older than 80 years between 2005 to 2015 [26]. Consequently, it is of great interest to know the outcomes of these old and very old patients after ICU care [1,2,5,7,27,28]. Some studies have demonstrated that although mortality is high (up to 60% one year after discharge), age itself it is not an independent risk factor for mortality [1,5,15]. Recently, in addition to mortality, other parameters such as functional status and quality of life after ICU discharge have also been evaluated [3,8,9,11,27,29]. However, most of these studies are mainly restricted to surgical patients, whereas medical patients are scarcely represented. Indeed, one study suggested that a medical condition is an independent factor for ICU refusal in patients aged 80 years or more [3]. Therefore, most published reports have a low proportion of medical patients, thereby making it difficult to achieve conclusions about the long-term outcomes of this specific group of elderly patients after ICU care.

In a small sample (*n *= 32) of medical patients Chelluri *et al*. [29] observed that 84% of patients independent for ADL prior to ICU entry maintained this situation whereas the quality of life improved slightly one year later. Montuclard [8] evaluated 28 medical patients ≥70 years with a long ICU stay (≥30 days) several months after discharge and observed increased dependence in some ADL (bathing, dressing, toileting, transfer and continence) and decreased quality of life in specific domains (global health, memory, sociability, leisure), in spite most of the patients remaining independent. In another study, Garrouste-Orgeas [3] evaluated 48 patients older than 80 years and observed no differences in ADL before and after ICU stay, although the quality of life was significantly worse in some domains (isolation, emotion, mobility) compared to a matched population. Kaarlola [9] evaluated a larger sample (*n *= 299) with a mailed-QOL questionnaire and detected that 88% of elderly survivors assessed their post ICU health status as good or satisfactory, 53% needed no assistance and one-third lived alone at home. Finally, De Rooij *et al*. [11] concluded that long-term elderly survivors after ICU care showed fair to good cognitive, functional and QOL status. However, only 5% (*n *= 11) of these subjects were medical patients and the ICU stay was too short (<5 days in 88% of patients). The main limitations of these studies were the sample size, retrospective data collection, absence of functional and QOL evaluation prior to ICU entry, high variability in demographic context and, in some instances, the use of non-validated scales to assess the functionality and quality of life of the subjects.

To avoid the limitations described above we have selected a well defined population of healthy community-dwelling elderly patients prior to ICU entry who required critical care for a medical condition and were alive one year after discharge.

We observed a low survival rate (49%) 12 months after discharge in this population, although the survivors had a relatively good health status in terms of functional and cognitive status as well as in the perceived quality of life. In this sense, 73%, 83% and 69% of patients showed similar scores in IADL and ADL autonomy and quality of life evaluation, respectively, compared to pre-ICU status. Interestingly, only one quarter of the patients with a Barthel Index lower than 50 at hospital discharge reached full recovery one year later. However, a major concern in these patients is the two-fold increase in the prevalence of main geriatric syndromes (mainly polypharmacy, urinary incontinence and depression) even at long-term follow-up that obviously reduced the perceived quality of life. On the other hand, patients with a short survival time (less tan one year) after ICU care were older, more frequently required mechanical ventilation and had worse scores at baseline (Lawton Index and EQ-5D vas) and also had more geriatric syndromes at baseline compared to patients with a long survival time. No differences were detected between the two groups in ICU scores, comorbidity, length of ICU stay and the main diagnoses at ICU admission or in functional status (Barthel Index) at hospital discharge. Our results firmly suggest that pre-clinical frailty before ICU care defined as a lower score in the Lawton Index may be a good marker to identify a population with a high risk of bad outcome after discharge.

Some mandatory questions arise from the present data: Is it possible to improve the outcomes of these elderly survivors after ICU care?, Could we introduce changes (for example: systematic multidisciplinary management) in the post-ICU follow-up of elderly patients to achieve better results?, Which subpopulation of medical elderly patients could benefit from specific interventions to improve outcomes after ICU care?

Only a recent study by Somme *et al*. [30] has tried to answer some of these questions. In a well designed prospective randomised clinical trial they compared the benefits of "geriatric care" versus "standard care" in the management of a small sample (*n *= 45) of subjects greater than 75 years old surviving a medical ICU admission. The main results showed no significant differences in the two study groups, although the outcomes in the "geriatric care cohort" were slightly better six months after discharge. However, these negative results must be considered with caution because the sample studied was very small and did not have enough statistical power, as was pointed out by the authors [30].

Our manuscript has several strengths such as the prospective enrolment of the subjects, long-term follow-up, inclusion of only medical patients with a good baseline health status and finally, the use of validated geriatric scales to objectively assess patient status. On the other hand, the main limitation is that the results can not be extrapolated to all elderly patients admitted to the ICU because we selected only medical patients with a good health status prior to ICU entry. Indeed, one third of the elderly patients admitted to our ICU did not fulfill the inclusion criteria and were excluded. Although our sample is not very large, it is quite homogeneous and representative of healthy community-dwelling elderly patients with a theoretical long life-expectancy before ICU admission and also with the best chance of survival to ICU care. Patient outcomes would probably be worse in a non-selected elderly population.

## Conclusions

In summary, in a well-selected population of healthy elderly people prior to critical care admission the expected outcomes in terms of survival 12 months after medical ICU discharge are bad because the mortality may be up to 51% of the subjects. However, the functional autonomy, cognitive status and quality of life were apparently good in the survivors, although a great increase in the prevalence of geriatric syndromes was observed. As a reflection of these results, most of the survivors (74%) would accept readmission to the ICU if necessary. It remains to be elucidated whether changes in the post-ICU management of these patients could improve their outcomes.

## Key messages

• Outcomes of previously healthy elderly patients after non-elective medical ICU admission were bad (one-year mortality was 50%, whereas functional autonomy and quality of life were significantly lower compared to baseline).

• A two-fold increase in geriatric syndromes after ICU care was observed.

• A Barthel Index (≥60) or EQ-5D _vas _(≥40) at hospital discharge were associated with full-functional recovery in most of the patients.

• It remains to be elucidated whether changes in the post-ICU management of these patients could improve their outcomes.

## Abbreviations

ADL: Activities of Daily Life; APACHE III: Acute Physiology and Chronic Health Evaluation III; BI: Barthel Index; CGA: Comprehensive Geriatric Assessment; CVVHDF: continuous venovenous haemodiafiltration; EQ-5D: EuroQol-5D; EQ-5D vas: Visual analogic scale of EuroQol-5D; IADL: Instrumental Activities of Daily Life; ICU: intensive care unit; IQCODE: Informant Questionnaire on Cognitive Decline in the Elderly; KI: Kappa Index; LI: Lawton Index; MMSE: minimental status evaluation; QOL: quality of life; SOFA: Sepsis-related Organ Failure Assessment.

## Competing interests

The authors declare that they have no competing interests.

## Authors' contributions

ES, JMN and AL-S contributed to study conception and design. PC and JMN contributed to recruitment and follow-up of the patients during their ICU stay. JMP-C, FM and MN contributed to recruitment and follow-up of the patients after their ICU stay. ES and JMP-C contributed to introduction of collected data in SPSS file for analysis. ES, JMN and AL-S contributed to analysis and interpretation of the data. ES, JMP-C and AL-S contributed to drafting of the article. ES, JMP-C, JMN, FM, MN, PC and AL-S contributed to critical revision and final approval of the manuscript.
